# The Association Between Grip Strength Measured in Childhood, Young- and Mid-adulthood and Prediabetes or Type 2 Diabetes in Mid-adulthood

**DOI:** 10.1007/s40279-020-01328-2

**Published:** 2020-08-19

**Authors:** Brooklyn J. Fraser, Leigh Blizzard, Marie-Jeanne Buscot, Michael D. Schmidt, Terence Dwyer, Alison J. Venn, Costan G. Magnussen

**Affiliations:** 1 Menzies Institute for Medical Research, University of Tasmania, Private Bag 23, Hobart, TAS 7001 Australia; 2 Department of Kinesiology, University of Georgia, Athens, USA; 3 George Institute for Global Health, Oxford Martin School and Nuffield Department of Obstetrics and Gynaecology, Oxford University, Oxford, UK; 4 Murdoch Children’s Research Institute, Melbourne, Australia; 5 Faculty of Medicine, Dentistry and Health Sciences, University of Melbourne, Melbourne, Australia; 6 Research Centre of Applied and Preventive Cardiovascular Medicine, University of Turku, Turku, Finland; 7 Centre for Population Health Research, University of Turku and Turku University Hospital, Turku, Finland

## Abstract

**Background:**

Although low child and adult grip strength is associated with adverse cardiometabolic health, how grip strength across the life course associates with type 2 diabetes is unknown. This study identified the relative contribution of grip strength measured at specific life stages (childhood, young adulthood, mid-adulthood) with prediabetes or type 2 diabetes in mid-adulthood.

**Methods:**

Between 1985 and 2019, 263 participants had their grip strength measured using an isometric dynamometer in childhood (9–15 years), young adulthood (28–36 years) and mid-adulthood (38–49 years). In mid-adulthood, a fasting blood sample was collected and tested for glucose and glycated haemoglobin (HbA1c). Participants were categorized as having prediabetes or type 2 diabetes if fasting glucose levels were ≥ 5.6 mmol or if HbA1c levels were ≥ 5.7% (≥ 39 mmol/mol). A Bayesian relevant life course exposure model examined the association between lifelong grip strength and prediabetes or type 2 diabetes.

**Results:**

Grip strength at each time point was equally associated with prediabetes or type 2 diabetes in mid-adulthood (childhood: 37%, young adulthood: 36%, mid-adulthood: 28%). A one standard deviation increase in cumulative grip strength was associated with 34% reduced odds of prediabetes or type 2 diabetes in mid-adulthood (OR 0.66, 95% credible interval 0.40, 0.98).

**Conclusions:**

Greater grip strength across the life course could protect against the development of prediabetes and type 2 diabetes. Strategies aimed at increasing muscular strength in childhood and maintaining behaviours to improve strength into adulthood could improve future cardiometabolic health.

**Video abstract:**

The Association Between Grip Strength Measured in Childhood, Young- and Mid-adulthood and Prediabetes or Type 2 Diabetes in Mid-adulthood

**Electronic supplementary material:**

The online version of this article (10.1007/s40279-020-01328-2) contains supplementary material, which is available to authorized users.

## Key Points

**Table:** 

This study is the first to use a life course model to identify the relative contribution of muscular strength measured at different stages across the life course to the development of type 2 diabetes in mid-adulthood. We found that muscular strength measured in childhood, young- and mid-adulthood was equally associated with prediabetes or type 2 diabetes in mid-adulthood.
These findings emphasize the importance of a life course approach to the prevention of type 2 diabetes and suggest that the health benefits of people of all ages participating in muscle-strengthening activities should be better promoted.
Strategies aimed at increasing muscular strength in childhood and maintaining behaviours to increase muscular strength into later life could be encouraged to help prevent the development of type 2 diabetes.

## Background

As the prevalence of type 2 diabetes and its precursor state of prediabetes increases [1], risk reduction strategies to prevent the development of this chronic disease are critical. A recent systematic review and meta-analysis of observational research presented in adults suggested that a one standard deviation increase in muscular strength is associated with a 13% lower risk of type 2 diabetes [2]. This finding is supported by a Mendelian randomization study that showed SNPs associated with higher grip strength, a proxy of muscular strength, to associate with lower odds of type 2 diabetes [3]. However, the link between muscular strength and impaired glucose homeostasis, a risk factor for type 2 diabetes, is not limited to adults. Greater childhood muscular strength is associated with lower levels of insulin resistance and beta cell function in adulthood [4, 5], while findings from a Swedish cohort of male military conscripts showed low muscular strength measured at age 18 years to associate with a 52% increased risk of type 2 diabetes 10–40 years later [6]. These findings suggest childhood muscular strength, often measured as grip strength, could be a potential early life target for strategies aimed at preventing type 2 diabetes in adulthood. However, previous observational studies have been limited to two time point analyses or had muscular strength data available only at baseline. It is currently unknown how grip strength measured across the life course is associated with type 2 diabetes.

Examining how grip strength associates with prediabetes or type 2 diabetes using life course approaches could inform future prevention strategies [7]. This association may be reflected by a critical period model, where grip strength at only one life stage is important for prediabetes or type 2 diabetes risk; a sensitive period model, where grip strength measured at one or more life stages has a greater effect on prediabetes or type 2 diabetes risk compared with grip strength at other life stages; or an accumulation model, where grip strength measured across the life course is equally important for the development of prediabetes or type 2 diabetes [7]. The pattern by which life course grip strength is associated with type 2 diabetes could provide insight into when interventions aimed at preventing type 2 diabetes by targeting muscular strength levels could be most effectively implemented.

This study aimed to identify the life course model that best describes the association between grip strength measured in childhood, young adulthood and mid-adulthood and the risk of prediabetes or type 2 diabetes in mid-adulthood.

## Research Design and Methods

### Participants

**Fig. 1:**
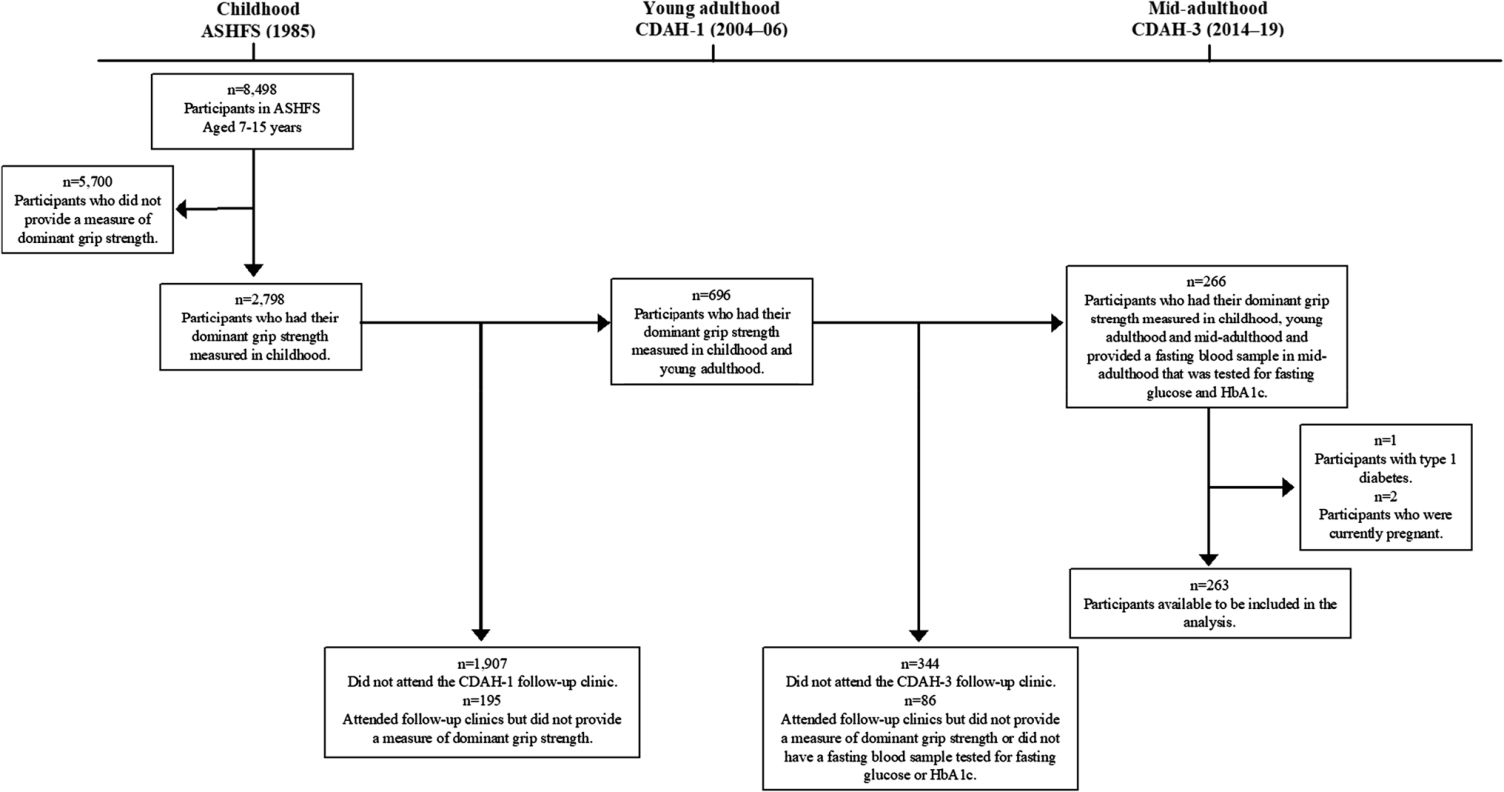
Flow chart of participation. *ASHFS* Australian Schools Health and Fitness Survey, *CDAH* Childhood Determinants of Adult Health, *HbA1c* glycated haemoglobin

In 1985, a nationally representative sample of Australian schoolchildren participated in the Australian Schools Health and Fitness Survey (ASHFS) and had their health and fitness assessed [8]. A subset of children aged 9, 12 and 15 years had their grip strength measured. Participants were followed up and attended clinics as part of the Childhood Determinants of Adult Health (CDAH) Study in 2004–06 when aged 28–36 years (young adulthood) and in 2014–19 when aged 38–49 years (mid-adulthood). During these adult follow-ups, participants had their grip strength reassessed and provided a fasting blood sample that was tested for glucose and glycated haemoglobin (HbA1c). Included in analyses were 263 participants who had their grip strength measured in childhood, young- and mid-adulthood and who provided a fasting blood sample in mid-adulthood, did not have type 1 diabetes and were not pregnant. A flowchart of participation is presented in Fig. 1. The ASHFS was approved by the State Directors General of Education. Follow-up studies were approved by the Southern Tasmania Health and Medical Human Research Ethics Committee and the Tasmania Health and Medical Human Research Ethics Committee. All participants provided written informed consent.

### Grip Strength Across the Life Course

In childhood, young- and mid-adulthood, right and left grip strength was measured by maximum voluntary contraction using an isometric dynamometer (Smedley’s Dynamometer, TTM, Tokyo, Japan) that was adjusted to fit the size of the participant’s hand. Grip strength was measured by participants gripping the dynamometer with maximum force with one hand, whilst the dynamometer rested on the opposite shoulder. In childhood, participants had one attempt at right and left grip strength. In adulthood, the maximum of two attempts was used in analyses. At each time point, participants reported whether their dominant hand was right or left. To remove the influence of body mass on grip strength performance, dominant grip strength not attributable to body mass at all three life stages was created by regressing dominant grip strength on body mass and using the residuals added to the grand mean [9], and standardized for age and sex.

### Anthropometric Measures

Regularly calibrated scales measured body mass to the nearest 0.5 kg in childhood and Heine scales (Heine, Dover, NH) measured body mass to the nearest 0.1 kg in adulthood. Height was measured to the closest 0.1 cm using a KaWe height tape (KaWe Kirchner & Wilhelm, Aspeg, Germany) in childhood and a Leicester height measure (Invicta, Leicester, UK) in adulthood. BMI was calculated as body mass (kg) divided by height (m) squared. Using a constant tension tape, child waist circumference was measured to the nearest 0.1 cm at the level of the umbilicus and adult waist circumference was measured at the narrowest point between the lower costal border and the iliac crest. Triceps, biceps, subscapular, and suprailiac skinfolds were measured using Holtain calipers (Holtain, Crymych, UK) to the nearest 0.2 mm in childhood and using Slim Guide Calipers to the nearest 0.5 mm in adulthood. Using the log of sum of four skinfolds, body density and fat percentage were calculated according to age-specific regression estimates [10]. Using the Siri formula, body fat was calculated from body density [11]. The difference between total body mass and fat mass was used to estimate fat-free mass.

### Cardiorespiratory Fitness

Cardiorespiratory fitness (CRF) was estimated as physical work capacity at 170 beats per minute (PWC_170_) using a Monark 818E bicycle ergometer (Monark Exercise AB, Vansbro, Sweden) in childhood, a Monark 828E bicycle ergometer (Monark Exercise AB, Vansbro, Sweden) in young adulthood and a Monark 928G3r bicycle ergometer (Monark Exercise AB, Vansbro, Sweden) in mid-adulthood. Participants pedalled at a cadence of 60 RPM and the test included three 3-min workloads (childhood) or three 4-min workloads (adulthood) that increased resistance stepwise. In the final minute of each workload, watts and heart rate were measured, and the regression lines were extrapolated to estimate PWC_170_. To remove the influence of muscle mass, measures of PWC_170_ not attributable to fat-free mass were created by regressing PWC_170_ on fat-free mass and using the residuals added to the grand mean.

### Prediabetes and Type 2 Diabetes

In mid-adulthood, participants provided a blood sample that was tested for glucose using a Siemens Advia 2400 Chemistry analyzer (Siemens Healthcare Diagnostics Inc., Deerfield, IL, USA) and HbA1c using a Bio-Rad D100 HbA1c analyzer (Bio-Rad Laboratories Inc., Hercules, CA, USA). A fasting status of ≥ 8 h was confirmed with the participant upon clinic arrival. Participants were categorized as having prediabetes or type 2 diabetes if they self-reported having type 2 diabetes or being on medication for type 2 diabetes, or if their fasting glucose levels were ≥ 5.6 mmol/L and/or HbA1c levels were ≥ 5.7% (≥ 39 mmol/mol), as defined by the American Diabetes Association [12].

### Statistical Analyses

#### Demographics

Participant characteristics were examined using Stata (Version 15.0, StataCorp, College Station, Texas). For continuous variables, mean and standard deviation (SD) are presented. For categorical variables, percentage and number of participants are reported.

#### Bayesian Model for Life Course Investigation

In R (Version 3.5.3, R Foundation for Statistical Computing, Vienna, Austria) [13] using the Stan package to fit Bayesian models [14], the Bayesian relevant life course exposure model (BRLM) was used to identify the relative importance of grip strength measured in childhood, young adulthood and mid-adulthood on prediabetes or type 2 diabetes in mid-adulthood [15, 16]. Full methodological detail outlining the BRLM has been published previously [15, 16] and is summarized in the supplementary material. Briefly, the relative importance of grip strength at each period to the development of prediabetes or type 2 diabetes is assumed by weights (childhood = W1, young adulthood = W2, mid-adulthood = W3), allowing grip strength to associate with prediabetes or type 2 diabetes at different levels depending on the life stage at which it was measured. The relative weights and the joint posterior distribution of the weight parameters at each of the three life stages, visualized using a ternary plot, help determine the life course model best supported by the data. When the posterior distribution of weights cluster along vertices of the ternary plot, the model indicates critical periods for the corresponding life stage, and when the posterior distribution clusters in the central area of the plot, the model suggests an accumulation model [15, 16]. The BRLM also estimates an overall effect for the lifetime exposure of grip strength, representing the maximum accumulated effect of grip strength across the life course on prediabetes or type 2 diabetes, and derives life stage-specific effects (a combination of the overall effect and relative weights), representing the time-dependent association between grip strength and prediabetes or type 2 diabetes [15, 16]. Posterior distributions were used to compute mean and 95% credible intervals (95% CrI) for weights (interpreted as relative importance) and odds ratios (OR) for the overall effect.

In a sensitivity analysis, lifetime average standardized values of CRF and waist circumference were included as covariates. These covariates were derived by age- and sex-standardizing CRF and waist circumference in childhood, young- and mid-adulthood and creating a numerical average from across the life course.

## Results

### Demographics

Participant characteristics are presented in Table 1. The average length of follow-up between childhood and mid-adulthood was 32.5 (1.1) years. Dominant grip strength and PWC_170_ were greatest in young adulthood. Body mass, waist circumference and fat-free mass increased with increasing age. Of the 263 participants, 48.3% (*n* = 127) were male and 7.2% (*n* = 19) had prediabetes or type 2 diabetes in mid-adulthood.

**Table 1:** Characteristics of participants

Characteristic	Childhood		Young adulthood		Mid-adulthood	
	*n*	Statistic*	*n*	Statistic*	*n*	Statistic*
Age (years)	263	11.7 (2.4)	263	31.6 (2.5)	263	44.2 (2.7)
Right grip strength (kg)	263	22.6 (8.6)	263	38.6 (10.4)	263	38.4 (10.7)
Left grip strength (kg)	263	22.0 (8.6)	262	36.7 (10.7)	263	36.4 (10.8)
Dominant grip strength (kg)	263	22.7 (8.6)	263	38.7 (10.6)	263	38.2 (10.7)
Dominant grip strength not attributable to body mass (kg)	263	23.0 (8.1)	263	39.0 (10.5)	263	38.2 (10.6)
Age- and sex-standardized dominant grip strength not attributable to body mass	263	0.08 (1.00)	263	0.05 (1.00)	263	–0.01 (1.07)
Body mass (kg)	263	41.9 (12.7)	263	74.8 (14.8)	263	80.0 (17.4)
Waist circumference (cm)	263	63.4 (7.4)	249	82.1 (10.6)	263	87.0 (12.3)
Body mass index (kg/m^2^)	263	18.2 (2.7)	262	24.9 (3.8)	263	26.8 (4.9)
Fat-free mass (kg)	263	32.8 (8.9)	248	53.9 (11.4)	260	55.2 (11.3)
PWC_170_ (W)	250	91.1 (35.9)	248	165.5 (50.1)	185	145.7 (66.8)
PWC_170_ not attributable to fat-free mass (W)	250	91.6 (32.6)	244	168.0 (47.1)	182	146.4 (65.7)
Fasting glucose (mmol/L)					257	4.7 (0.5)
HbA1c (%)					263	5.2 (0.3)
Prediabetes or type 2 diabetes					263	
No						92.8% (244)
Yes						7.2% (19)

*Mean (standard deviation) for continuous variables and percentage (number of participants) for categorical variables

*HbA1c* glycated haemoglobin, *PWC*_*170*_ physical work capacity at 170 beats per minute

### Bayesian Model for Life Course Investigation

**Fig. 2:**
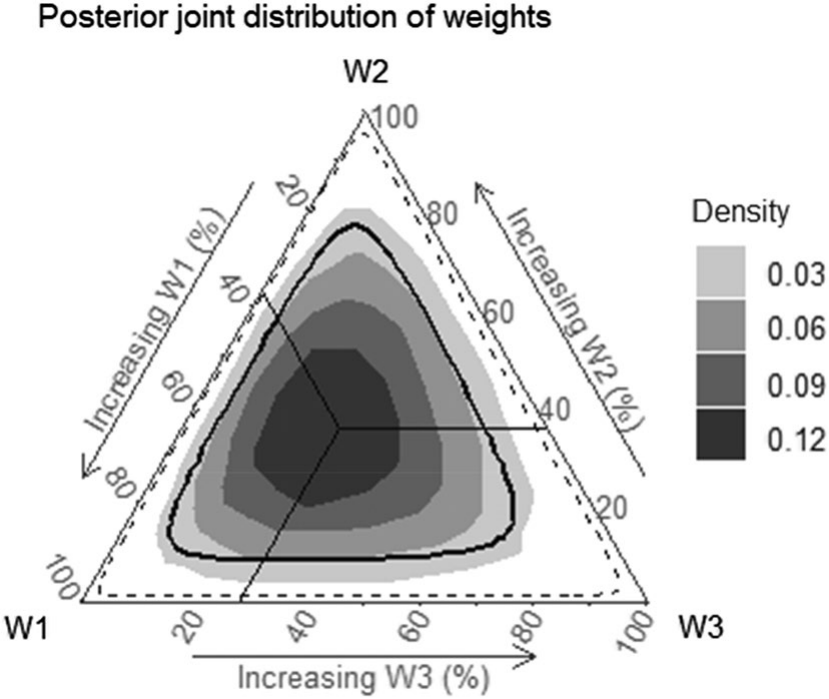
Posterior joint distribution of weights estimated for dominant grip strength measured in childhood, young adulthood and mid-adulthood with 50% (thick solid line) and 95% (dashed line) credible intervals. The solid lines represent the mean posterior probability of the weights. Darker areas represent a higher density of posterior mean estimates of the weights. The location of the posterior joint distribution of weights highlights the life course model best supported by the data. For example, in a critical period model the highest density of posterior mean estimates of the weights would lie along one of the vertices (W1 = childhood critical period; W2 = young adulthood critical period; W3 = mid-adulthood critical period); in an accumulation model, the highest density of posterior mean estimates of the weights would be around the central point; and in a sensitive period model the highest density of posterior mean estimates of the weights would be between the vertices and the central point. *W1* posterior mean estimates of weights for childhood, *W2* posterior mean estimates of weights for young adulthood, *W3* posterior mean estimates of weights for mid-adulthood

**Fig. 3:**
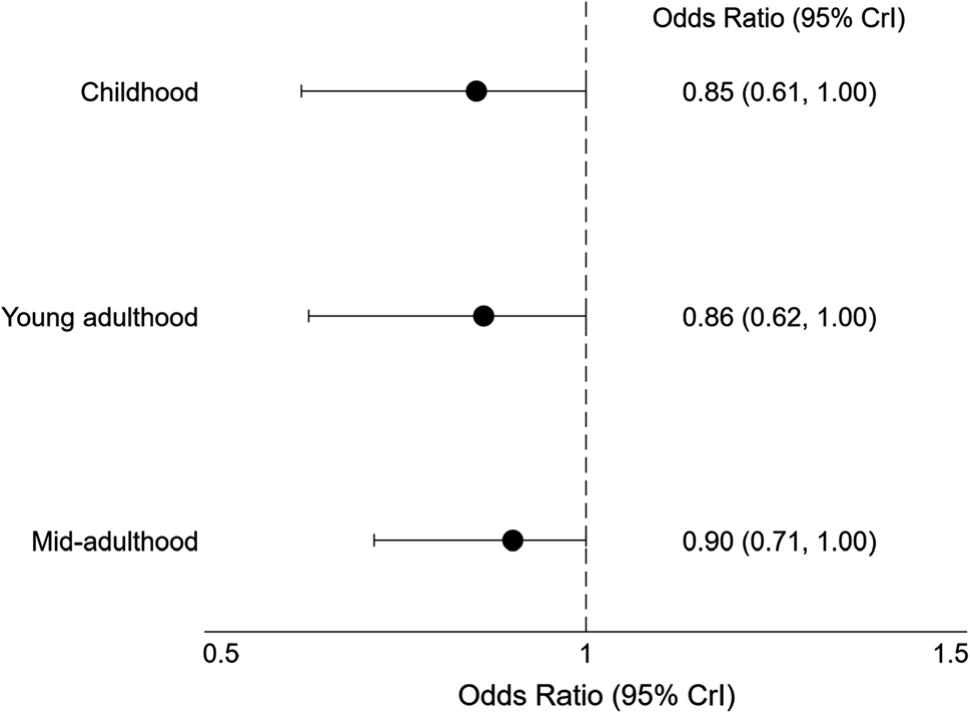
Life stage-specific associations between dominant grip strength and prediabetes or type 2 diabetes. *CrI* credible intervals

The unadjusted association between life course grip strength and prediabetes or type 2 diabetes in mid-adulthood is presented in Table 2. As reflected by the lifetime effect, a one SD increase in life course cumulative grip strength was associated with 34% reduced odds of prediabetes or type 2 diabetes in mid-adulthood (OR 0.66, 95% CI 0.40, 0.98). Contributing to this lifetime effect was grip strength measured in childhood, young- and mid-adulthood. The relative importance of grip strength measured at each of these life stages in relation to the odds of developing prediabetes or type 2 diabetes in mid-adulthood was approximately equal (ranging from 28 to 37%; Table 2 and Fig. 2), although the posterior (Fig. 2) and prior distributions (Figure S1) overlapped. As the relative importance values (i.e. weights) were not exactly equal, the association between life course grip strength and prediabetes or type 2 diabetes was suggestive of a relaxed accumulation life course model (W1 ≈ W2 ≈ W3) compared with a pure accumulation model (W1 = W2 = W3). The life stage-specific effects, derived as the product of the lifetime effect and life stage-specific relative weights, presented in Fig. 3 support this interpretation. A one SD increase in grip strength at each examined life stage was similarly associated with prediabetes or type 2 diabetes in mid-adulthood.

**Table 2:** Association between dominant grip strength and prediabetes or type 2 diabetes

	Odds ratio (95% CrI)	Relative importance (95% CrI)
Prediabetes or type 2 diabetes		
Lifetime effect	0.66 (0.40, 0.98)	
Life stages		
Childhood		37% (4%, 78%)
Young adulthood		36% (3%, 78%)
Mid-adulthood		28% (2%, 69%)

*CrI* credible intervals

When life course CRF and waist circumference were included in the model as covariates, evidence for a relaxed accumulation model persisted, with the relative importance of grip strength at each life stage remaining essentially unchanged (Table S1). The lifetime effect and each life stage-specific effect attenuated, and statistical significance was lost, although the effects remained in a protective direction (Tables S1 and S2).

## Discussion

This study is the first to identify the relative contribution of grip strength measured across the life course with prediabetes or type 2 diabetes in mid-adulthood. Our estimates suggest an approximately equal contribution from grip strength measured in childhood, young adulthood and mid-adulthood on prediabetes or type 2 diabetes risk and that greater cumulative grip strength across the life course was associated with a 34% reduction in the odds of developing prediabetes or type 2 diabetes in mid-adulthood. These findings are consistent with recent confirmation of a causal link between grip strength and type 2 diabetes from Mendelian randomization analysis [3] by demonstrating the cumulative nature of the association across the life course. As such, our data support the importance of developing and maintaining higher levels of muscular strength beginning in childhood and continuing through mid-adulthood to maximize future cardiometabolic health benefits.

Despite this being the first study to apply a life course modelling framework to examine the association of grip strength with prediabetes or type 2 diabetes, previous work provides a strong rationale for a causal link. For example, a recent two-sample Mendelian randomization study [3] that applied SNPs associated with grip strength obtained from the UK Biobank to data from two large meta-analysis consortia of type 2 diabetes and glycaemic traits (DIAGRAM and MAGIC) found a one SD increase in grip strength was associated with 23% lower odds of type 2 diabetes (OR 0.77, 95% CI 0.62, 0.95) [3]. The association between measures of muscular strength and type 2 diabetes risk is also supported by observational data. Results from a systematic review and meta-analysis suggest that in adulthood, a one SD increase in muscular strength is associated with a 13% decreased risk of type 2 diabetes (RR 0.87, 95% CI 0.81, 0.94), independent of BMI or waist to hip ratio [2]. The longitudinal association between child and adolescent muscular strength with adult type 2 diabetes and associated risk factors has also been described, independent of CRF and waist circumference. Higher levels of childhood muscular strength were associated with lower adult levels of insulin resistance and beta cell function, precursors of type 2 diabetes, among cohorts from Australia and Europe [4, 5], while low levels of muscular strength among Swedish male military conscripts aged 18 years were associated with an increased risk of type 2 diabetes 10–40 years later, independent of CRF and BMI [6].

Our findings expand current evidence by suggesting grip strength measured at three life stages were similarly associated with prediabetes or type 2 diabetes in mid-adulthood. Consequently, childhood, young adulthood and mid-adulthood are equally important life stages that can be targeted to help protect against the development of type 2 diabetes. That is, it is not grip strength at a single period in the life course or the tracking of grip strength from distal to proximal time points that explains the association with type 2 diabetes. These results suggest that greater cumulative grip strength across the life course is important in preventing prediabetes and type 2 diabetes. These findings support current national and global physical activity guidelines where both children and adults are encouraged to participate in muscle-strengthening activities [17, 18]. Strategies aimed at promoting environments and factors leading to muscular strength gains in childhood and initiating and maintaining participation in muscle-strengthening activities into adulthood could help prevent the development of type 2 diabetes. Evidence suggests resistance training interventions administered in schools can increase childhood muscular fitness levels [19]. Furthermore, the modifiable factors of lower adiposity and higher fat-free mass, CRF, flexibility, and speed capability could be targeted for strategies aimed at increasing childhood muscular strength [20]. Of concern, childhood muscular fitness levels have declined over time [21], and as muscular strength tracks between childhood and adulthood [22], this decline could have long-term effects on future muscular strength. Therefore, implementing well-informed strategies aimed at improving muscular strength in childhood are required to help promote favourable muscular strength levels across the life course.

The mechanism explaining the association between grip strength and type 2 diabetes is unknown. Associations may be acting indirectly through adiposity levels. However, grip strength appears to associate with type 2 diabetes independent of adiposity levels. The direct association between grip strength and type 2 diabetes could be explained by resistance training-induced improvements in glucose homeostasis, whereby resistance training lowers HbA1c levels [23, 24] and upregulates key proteins in the insulin signalling cascade [25]. Given grip strength is a measure of overall muscular strength [26] and resistance training increases muscular strength levels [27, 28], the glucose homeostasis benefits of resistance training are likely to explain the observed association. However, whether the link between behaviours that increase muscular strength and type 2 diabetes explain the relaxed accumulation model highlighted in this study is unknown. Genetic factors or the persistence of higher levels of fat-free mass and protective innate muscle traits, such as mitochondrial density, intramuscular fat and skeletal muscle fibre type, across the life course could be responsible for the accumulative effect of muscle strength on type 2 diabetes risk. Although additional research is required to confirm the exact mechanisms, results from this study suggest protective effects begin in childhood and that greater cumulative grip strength across the life course is beneficial. These data reinforce the causal link between grip strength and type 2 diabetes highlighted by Mendelian randomization analysis [3].

This study had limitations. Due to time and economic constraints at baseline, a subset of children had grip strength measured. For inclusion in our analysis dataset, those with grip strength measured at baseline had to attend and pass fitness exclusion questionnaires at both follow-ups. A substantial proportion did not fulfil all participation requirements, resulting in a relatively small sample size and case numbers for analysis. Nevertheless, our simulation study showed that we had > 80% power to detect the true life course model in a sample of this size given the prevalence of type 2 diabetes (see supplementary material). Whilst we cannot discount participation bias, it is reassuring that participants and non-participants were similar in baseline characteristics, and that for the two characteristics (socioeconomic status and smoking status) in which there were differences, the strength of the inverse relationship between baseline grip strength and prediabetes or type 2 diabetes for non-participants at follow-up was close to uniform in each category of socioeconomic status and smoking status. In all categories of socioeconomic status and for the large group of non-smokers, our estimate of the inverse relationship between baseline grip strength and risk of prediabetes or type 2 diabetes was stronger (further from the null) for non-participants than for participants. This raises the possibility that the protective effect of grip strength has been underestimated in this study (any bias is towards the null). Furthermore, given our low sample size, the posterior (Fig. 2) and prior distributions (Figure S1) overlapped and credible intervals were wide. We recommend that the research be replicated in other cohorts with larger sample sizes to see if findings are consistent. Furthermore, the newly developed BRLM approach does not currently allow inclusion of time varying covariates. In our study, it was important to attempt to remove the influence of CRF and waist circumference from the association between life course grip strength and prediabetes or type 2 diabetes. In the absence of a formal method incorporated within the BRLM, we included an average life course standardized value of CRF and waist circumference in the model. Although this approach is not ideal as it considers a cumulative effect averaged from one to three time points, this was the best approach available to us. Strengths of this study include the use of a national cohort including both sexes with a baseline age of 9–15 years and a follow-up period of over 30 years. Furthermore, measures of grip strength were available at three time points across the life course. This meant the BRLM could be used to address a research question that was, up to this point, unknown. Lastly, grip strength, a measure of overall muscular strength [26], is a reliable and valid field-based measure [29] and correlates with the one repetition maximum, a gold standard test to assess muscular strength [30].

## Conclusion

Notwithstanding the limitations outlined, these findings suggest that grip strength measured in childhood, young adulthood and mid-adulthood was equally associated with prediabetes or type 2 diabetes highlighting the importance, and future cardiometabolic health benefits, of greater cumulative grip strength across the life course. Implementing strategies aimed at increasing muscular strength in childhood and maintaining these behaviours into later life could help protect against the development of prediabetes and type 2 diabetes.

## Electronic supplementary material

Below is the link to the electronic supplementary material.

Supplementary material 1 (DOCX 93 kb)

## Declarations

### Data availability statement

The datasets generated during and/or analysed during the current study are available from the corresponding author on reasonable request.
