# Supplementary material for: The Association Between Grip Strength Measured in Childhood, Young- and Mid-adulthood and Prediabetes or Type 2 Diabetes in Mid-adulthood
Source: Sports Med. 2020 Aug 19;51(1):175–83. doi: 10.1007/s40279-020-01328-2 (PMC7806554; doi:10.1007/s40279-020-01328-2)
Supplement: Supplementary file 2 — Supplementary material 1 (DOCX 93 kb) [file 40279_2020_1328_MOESM1_ESM.docx]

**Supplementary material**

Title: The association between grip strength measured in childhood, young- and mid-adulthood and prediabetes or type 2 diabetes in mid-adulthood

Journal: *Sports Medicine*

Authors and affiliations:

Brooklyn J. Fraser, BBiotechMedRes(Hons) *^a^*

Leigh Blizzard*, PhD *^a^*

Marie-Jeanne Buscot*, PhD *^a^*

Michael D. Schmidt, PhD *^b^*

Terence Dwyer, MD, MPH *^a, c, d, e^*

Alison J. Venn, PhD *^a^*

Costan G. Magnussen, PhD *^a^*^,^ *^f, g^*

*^a^ Menzies Institute for Medical Research, University of Tasmania, Hobart, Tasmania, Australia.*

*^b^ Department of Kinesiology, University of Georgia, Athens, USA.*

*^c^ George Institute for Global Health, Oxford Martin School and Nuffield Department of Obstetrics & Gynaecology, Oxford University, Oxford, UK.*

*^d^ Murdoch Children’s Research Institute, Melbourne, Australia.*

*^e^ Faculty of Medicine, Dentistry and Health Sciences, University of Melbourne, Melbourne, Australia.*

*^f^ Research Centre of Applied and Preventive Cardiovascular Medicine, University of Turku, Turku, Finland.*

*^g^ Centre for Population Health Research, University of Turku and Turku University Hospital, Turku, Finland.*

* These two authors contributed equally to this work and are considered equal second authors.

Correspondence to Costan G Magnussen, Menzies Institute for Medical Research, University of Tasmania, Private Bag 23, Hobart 7001, Tasmania, Australia. E-mail: [cmagnuss@utas.edu.au](mailto:fraserbj@utas.edu.au)

Supplementary methods and results

*Additional detail regarding the Bayesian relevant life course exposure model (BRLM) and our simulation study to assess the performance of the BRLM under small sample size and low outcome frequency.*

The relative importance of grip strength measured in childhood, young adulthood and mid-adulthood on prediabetes or type 2 diabetes risk in mid-adulthood is identified using a Bayesian relevant life course exposure model (BRLM) [1, 2]. The BRLM assumes weights for the effect of grip strength at different life stages (childhood=W1, young adulthood=W2, mid-adulthood=W3), allowing grip strength to associate with prediabetes or type 2 diabetes differently depending on the life stage at which it was measured. The BRLM considers a model of weighted exposure for each considered life stage, conceptualized as the product of the exposure metric and its corresponding weight over each considered life stage, summed over all life stages. The weight relates to the relevance of grip strength during each period to the development of prediabetes or type 2 diabetes in mid-adulthood. The life stage specific weight parameters are estimated using a Bayesian approach. The estimated weights help determine the life course model best supported by the data. The joint posterior distribution of the weight parameters at each of the three life stages can be visualized using a ternary plot. When the posterior distribution of weights cluster along vertices, the model indicates critical periods for the corresponding weight stage, and when the posterior distribution clusters in the central area of the plot, the model suggests an accumulation model [1, 2]. In addition, the BRLM estimates an overall effect for the lifetime exposure of grip strength, representing the maximum accumulated effect of grip strength across the life course on prediabetes or type 2 diabetes in mid-adulthood. Furthermore, the BRLM method derives life stage specific effects, a combination of the overall effect and relative weights. These represent the time dependent association between grip strength (in childhood, young adulthood and mid-adulthood) and prediabetes or type 2 diabetes in mid-adulthood [1, 2].

In R (Version 3.5.3, R Foundation for Statistical Computing, Vienna, Austria) [3] using the Stan package to fit Bayesian models [4], the BRLM with an unconditional logistic regression likelihood was used to identify the relative importance of grip strength measured in childhood, young adulthood and mid-adulthood on prediabetes or type 2 diabetes. There was little or no evidence to include prior beliefs on what life course model would best support these data. Therefore, a non-informative prior, giving equal support to all life models, was used. In this study, a non-informative Dirichlet (1, 1, 1) prior for weights and a Cauchy prior (0, 2.5) for the lifetime effect were used. Posterior distributions were used to compute mean and 95% credible intervals (95% CrI) for weights (interpreted as relative importance) and OR for the overall effect. Measures of CRF and waist circumference in childhood, young adulthood and mid-adulthood were age- and sex-standardized and a numerical average from across the life course, representing an average standardized value, was created ((z-childhood + z-young adulthood + z-mid-adulthood)/3). If data were not available at all three time-points, an average standardized value was created using the data that were available (one time-point: CRF: n=11; waist circumference: n=0; two time-points: CRF: n=91; waist circumference: n=14; three time-points: CRF: n=163; waist circumference: n=251). In a sensitivity analysis, the lifetime average of standardized CRF and waist circumference values were included within the model as covariates using Cauchy priors (0, 2.5) for each continuous covariate.

*Simulation study to assess the performance of the BRLM approach under small sample size and low outcome frequency*

Since the BRLM is a recently developed approach, its performance at identifying the correct life course model by estimating ‘true’ parameter values has not been investigated in a scenario including a small sample size (<300 participants) and a rare binary outcome (frequency <10 %). To assess the performance of the model to predict a pure accumulation model in a dataset matching this scenario, we simulated a three-period pure accumulation (W1=W2=W3=0.33) life course model (‘true’ model), with three exposure gaussian distributed variables with a correlation of 0.75 between adjacent measures and 0.50 between non-adjacent measures. The parameter for the lifetime effect was set to beta=2 and for simplicity, covariates were not included. Using this model, the binary dependent variable was simulated for a sample of size N=265 using an inverse logit function of the cross product of the lifetime effect multiplied by the weighted simulated exposure to approximately match the outcome prevalence with the prevalence of prediabetes or type 2 diabetes observed in our study (i.e. 7.5 %). This was repeated 5,000 times, and 5,000 random simulated samples of N=265, where the data generating process matched a pure accumulation scenario and the occurrence of the observed outcome was rare (<10 %), were produced. For each of these 5,000 simulated datasets, using the non-informative priors described in the previous section, the BRLM was fitted using 10000 iterations for burn-in and 20000 iterations for parameter inference to: (1) retrieve the posterior distribution for each of the three weight parameters, and (2), identify the shortest Euclidian distance between the estimated weights and five possible reference weight vectors corresponding to various life course models. These included one pure accumulation (where all three weights=0.33), three critical periods (i.e. where one of the three weights=1 and the other two=0), and one sensitive period (where the most weight was set to period 1 (0.75), with 20 % of the weight allocated to period 2 and 5 % to period 3, respectively), as previously described [1]. The shortest Euclidian distance represents the life course model best supported by the data.

*Results of the simulation study*

Across the 5,000 generated small (N=265) datasets, the average posterior mean of weights were close to the ‘true’ weights set in the simulation (i.e. mean (W1)=0.28, mean (W2)=0.27, mean (W3)=0.31) but the standard deviation around the means were relatively wide (i.e. SD (W1)=0.57, SD (W2)=0.62, SD (W3)=0.71). This suggest that although on average the BRLM estimates the correct weight values in a situation of small sample size such as ours, there is some variability around those estimates. However, using the Euclidian distance approach, the correct life course model (i.e. ‘pure’ accumulation) was identified for N=4,162/5,000 simulated samples with a sample size of N=265, suggesting that the BRLM has a power of 83.2 % at detecting the true data generating-process in situation of very small sample size and low disease prevalence.

**Supplementary references:**

1. Madathil S, Joseph L, Hardy R, Rousseau MC, Nicolau B. A Bayesian approach to investigate life course hypotheses involving continuous exposures. Int J Epidemiol. 2018 Oct 1;47(5):1623-35.

2. Madathil S, Blaser C, Nicolau B, Richard H, Parent ME. Disadvantageous socioeconomic position at specific life periods may contribute to prostate cancer risk and aggressiveness. Front Oncol. 2018;8:515.

3. R Core Team. R: A language and environment for statistical computing. Vienna, Austria: R Foundation for Statistical Computing; 2018.

4. Carpenter B, Gelman A, Hoffman MD, Lee D, Goodrich B, Betancourt M, et al. Stan: A probabilistic programming language. J Stat Softw. 2017;76(1).

| Figure S1. Distribution of the non-informative Dirichlet (1,1,1) prior with 50% (thick solid line) and 95% (dashed line) credible intervals. |  |
| --- | --- |
| 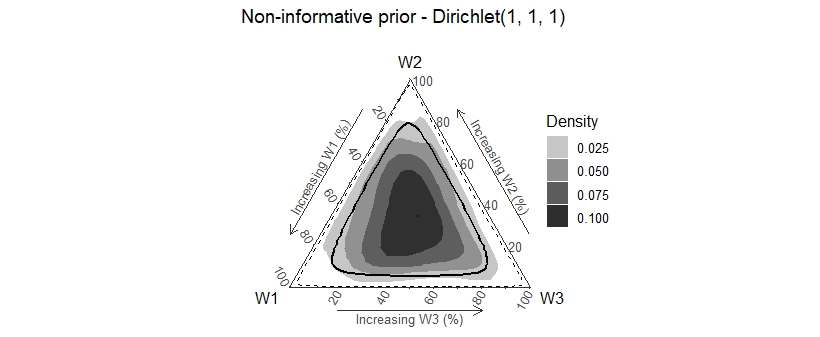 | |
| Abbreviations: W1, posterior mean estimates of weights for childhood; W2, posterior mean estimates of weights for young adulthood; W3, posterior mean estimates of weights for mid-adulthood. |  |

| Table S1. Association between dominant grip strength and prediabetes or type 2 diabetes adjusted for life course average standardized values of cardiorespiratory fitness and waist circumference. | | | | |
| --- | --- | --- | --- | --- |
|  | | Odds Ratio  (95% CrI) |  | Relative importance  (95% CrI) |
| Prediabetes or type 2 diabetes | |  |  |  |
| Life time effect | | 0.75 (0.45, 1.13) |  |  |
| Life stages | |  |  |  |
|  | Childhood |  |  | 34 % (3 %, 77 %) |
|  | Young adulthood |  |  | 37 % (4 %, 80 %) |
|  | Mid-adulthood |  |  | 29 % (2 %, 72 %) |
| Abbreviations: CrI, Credible Intervals. | | | | |

| Table S2. Life stage specific associations between dominant grip strength and prediabetes or type 2 diabetes adjusted for life course average standardized values of cardiorespiratory fitness and waist circumference. | |
| --- | --- |
|  | Odds Ratio (95% CrI) |
| Childhood | 0.90 (0.67, 1.03) |
| Young adulthood | 0.89 (0.65, 1.03) |
| Mid-adulthood | 0.92 (0.74, 1.03) |
| Abbreviations: CrI, Credible Intervals. | |
